# An In Vivo Confocal Microscopic Study of Corneal Nerve Morphology in Unilateral Keratoconus

**DOI:** 10.1155/2016/5067853

**Published:** 2016-01-21

**Authors:** Natasha Kishore Pahuja, Rohit Shetty, Rudy M. M. A. Nuijts, Aarti Agrawal, Arkasubhra Ghosh, Chaitra Jayadev, Harsha Nagaraja

**Affiliations:** ^1^Narayana Nethralaya Eye Hospital, Bangalore 560010, India; ^2^Academic Hospital, Maastricht University, P.O. Box 616, 6200 MD Maastricht, Netherlands

## Abstract

*Purpose.* To study the corneal nerve morphology and its importance in unilateral keratoconus.* Materials and Methods.* In this prospective cross-sectional study, 33 eyes of 33 patients with keratoconus in one eye (Group 3) were compared with the other normal eye of the same patients (Group 2) and 30 eyes of healthy patients (Group 1). All patients underwent detailed ophthalmic examination followed by topography with Pentacam HR and in vivo confocal microscopy (IVCM). Five images obtained with IVCM were analyzed using an automated CCmetrics software version 1.0 for changes in subbasal plexus of nerves.* Results.* Intergroup comparison showed statistically significant reduction in corneal nerve fiber density (CNFD) and length (CNFL) in Group 3 as compared to Group 1 (*p* < 0.001 and *p* = 0.001, resp.) and Group 2 (*p* = 0.01 and *p* = 0.02, resp.). Though corneal nerve fiber length, diameter, area, width, corneal nerve branch density, and corneal total branch density were found to be higher in decentered cones, only the corneal nerve branch density (CNBD) was found to be statistically significant (*p* < 0.01) as compared to centered cones.* Conclusion.* Quantitative changes in the corneal nerve morphology can be used as an imaging marker for the early diagnosis of keratoconus before the onset of refractive or topography changes.

## 1. Introduction

Keratoconus (KC) is characterized by stromal thinning and protrusion that leads to irregular astigmatism and altered optical performance of the cornea [1]. It has traditionally been described as a bilateral asymmetric disorder of the cornea though there are some reports on unilateral keratoconus [1–5]. The estimated frequency of the unilateral disease has been reported to range from 14.3% to 41% [1, 3]. Studying unilateral KC provides a comparative insight into disease pathogenesis as the unaffected fellow eye acts as an ideal control for the affected one with other contributing factors like atopy, genetics, and environment remaining constant for both [4, 5]. There is significant data on the management of keratoconus [6–8], with scarce literature on the mechanistic model of the disease itself. Nonetheless, abnormalities have been documented in all layers of keratoconic corneas [9, 10].

Recently, a review by Shaheen et al. [11] has highlighted the role of corneal nerves in health and disease of the cornea. A loose plexus of nerves under the Bowman's layer, formed by branches arising from the trigeminal nerve, is perforated to form the subbasal nerve plexus, where fibers of which terminate within the superficial epithelial cells as free nerve endings [12, 13]. Corneal nerves are known to regulate multiple pathways, which play crucial roles in several conditions including KC [11]. Significant changes in the corneal subbasal nerve plexus such as increased tortuosity, reduced nerve fiber, and branch density have previously been demonstrated in several diseases involving the cornea [10, 14].

Etiopathogenesis of KC has so far been studied in corneal buttons excised during penetrating keratoplasty as there are currently no animal models to study these cellular and morphological changes in vivo [15]. Corneal buttons excised from keratoconus patients represent advanced disease. It is therefore not possible to elucidate vital information, which may be seen in early disease. In vivo confocal microscopy (IVCM), a noninvasive imaging modality, has overcome this limitation and allows in vivo examination of the human cornea at a microstructure level [14, 16]. While there are studies of IVCM in bilateral KC [9, 16], there are none in patients with unilateral disease. We therefore evaluated the alterations in subbasal nerves with the IVCM in a cohort of unilateral KC patients. The aim of this study was to gain more insight into the role of corneal nerves in pathogenesis and diagnosis and as a marker for disease progression in KC.

## 2. Materials and Methods

The protocol of this prospective cross sectional study was approved by the hospital's ethics committee and was performed according to the tenets of the Declaration of Helsinki. Written informed consent was obtained from each patient after a detailed explanation about the nature of the study.

Thirty healthy subjects who did not show any evidence of KC on topography were taken as controls (Group 1). Sagittal curvature map on topography showing a localized area of increased keratometry, inferior-superior asymmetry, and skewed steep radial axis above and below the horizontal meridian were diagnosed to have KC. Thirty-three patients showing these changes in only one eye were included in the study. Group 2 included the normal eye (thirty-three eyes) and Group 3 included the keratoconic eye of these patients. Group 3 was further subdivided into centered cones (Group 3a) and decentered cones (Group 3b) based on whether the cones were located within the central 2 mm or beyond [17]. Patients with a history of contact lens use, ocular surgery, trauma, any coexisting corneal disease, evidence of corneal scarring, or bilateral keratoconus were excluded from the study.

All patients underwent a complete ophthalmic examination including refraction (uncorrected and corrected visual acuity), retinoscopy, detailed slit lamp evaluation, corneal topography using Pentacam HR Scheimpflug imaging system (Oculus Optikgerate GmBH, Wetzlar Germany), and laser-scanning IVCM using the Rostock Corneal Module/Heidelberg Retina Tomograph II (Heidelberg Engineering GmbH, Dossenheim, Germany). Both eyes of the unilateral keratoconus patients and only one eye of the control group underwent IVCM. The laser in vivo confocal microscope uses a diode laser of 670 nm wavelength. Proparacaine 0.5% drops were used to anesthetize the cornea. Patients were asked to fixate on a distance target aligned to enable examination of the central cornea. For each IVCM examination, five high quality clear images of the subbasal nerve plexus were chosen. A full 400 × 400 micron square frame was used for the analysis. After the procedure, one drop of 0.5% moxifloxacin eye drops was instilled to prevent any secondary infection.

**Figure 1:**
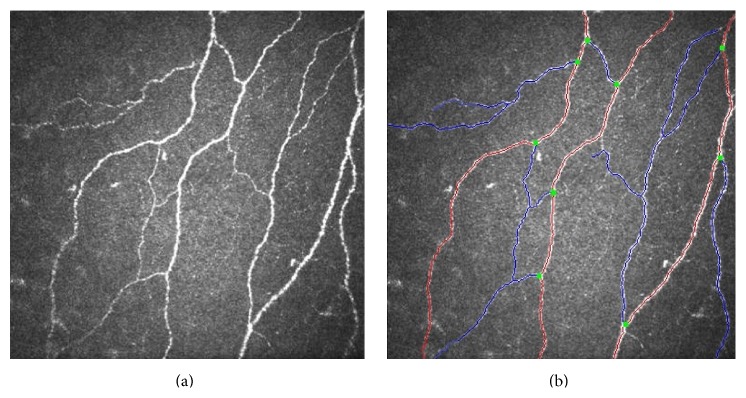
In vivo confocal microscopy image (a) of the subbasal plexus of nerves in a patient of keratoconus and the same image after analysis by the automated CCmetrics software (b).

The subbasal nerve plexus was quantitatively analyzed using an automated CCmetrics software version 1.0 (University of Manchester, UK) (Figure 1). A total of six parameters were quantified in all three groups for the analysis:

- corneal nerve fiber density (CNFD): the number of nerve fibers per mm^2^,
- corneal nerve branch density (CNBD): the number of branch points on the main nerve fibers per mm^2^,
- corneal nerve fiber length (CNFL): the total length of nerve per mm^2^,
- corneal total branch density (CTBD): the total number of branch points per mm^2^,
- corneal nerve fiber area (CNFA): the total nerve fiber area per mm^2^,
- corneal nerve fiber width (CNFW), the average nerve fiber width per mm^2^.

All images were acquired and analyzed by a single observer who was masked about the study groups. Data was analyzed and compared between the three groups and subgroups.

### 2.1. Statistical Analysis

Statistical analyses were performed using Stata version 12.1 (StataCorp, College Station, TX, USA) statistical software. The continuous variables were described using mean and standard deviation. The *t* test was used to compare the parameter values within the groups. *p* value <0.05 was considered statistically significant.

## 3. Results

A total of 480 images of subbasal plexus were analyzed with hundred and fifty images of Group 1, 165 images of Group 2, and 165 images of Group 3. Mean age of Group 1 was 28.06 ± 2.41 years and for Groups 2 and 3 it was 22.21 ± 4.66 years. Within Group 3, 18 eyes in Group 3 had centered cones and 15 eyes had decentered cones.

Table 1 shows the IVCM findings of different subbasal nerve parameters and the comparison between each group. The CNFD was 30.51 ± 5.8 mm/mm^2^ in Group 1, 28.48 ± 23.82 mm/mm^2^ in Group 2, and 23.82 ± 8.02/mm^2^ in Group 3. The lower density of nerves was statistically significant in eyes with KC when compared to Group 2 (*p* < 0.001) and Group 3 (*p* = 0.01). The CNFL also followed a similar pattern being 17.59 ± 3.16 mm/mm^2^ in Group 1, 16.6 ± 2.42 mm/mm^2^ in Group 2, and 14.82 ± 3.61 mm/mm^2^ in Group 3. The reduction in the fiber length was significant in Group 3 when compared to Group 2 (*p* = 0.02) and controls (*p* < 0.001). The mean value of CNBD, CTBD, and CNFA did not differ significantly within the groups (*p* = 0.14), (*p* = 0.23), and (*p* = 0.13), respectively. The mean CNFW was unaffected.

Group 3 was further subclassified and analyzed based on the location of cones (Table 2). The CNBD was 29.6 ± 18.9 mm/mm^2^ in Group 3a (centered cones) and 47.1 ± 16.7 mm/mm^2^ in Group 3b (decentered cones), which was a statistically significant difference (*p* < 0.01). The CNFD, CNFL, CTBD, CNFA, and CNFW were higher in Group 3b, but this was not statistically significant.

## 4. Discussion

With KC being a progressive disease, the changes in refractive and topographic parameters that help us decide further management for our patients have been defined [18]. Progression has also been documented using ultra high-resolution optical coherence tomography to detect changes in the Bowman's layer thickness. The Bowman's ectasia index (BEI) has been proposed as a sensitive qualitative and quantitative diagnostic tool [19]. However, all these indices are reliable only in established cases of KC. In cases of a unilateral KC, more than half of the normal fellow eyes develop KC within 16 years with majority of them manifesting in the first six years of followup [20]. Currently, there are no devices or investigative modalities that are capable of predicting this change [5]. With a varying degree of progression, the need for better and sensitive tools is warranted.

Microstructure and in vitro studies with light and electron microscope have shown changes in all layers of the cornea in KC [20, 21]. In vivo studies suffer from a limitation of inadequate resolution. With the introduction of the IVCM, it has now become possible to visualize all the layers of the cornea down to the resolution of a few microns, at various depths and at the cellular level [11, 22]. It has previously been used to study the normal corneal architecture [11, 16, 22], alterations in various diseases such as corneal infections caused by viruses [23], acanthamoeba [24], or fungi [25] and in inflammatory conditions like dry eye [26]. Studies on corneal nerves in chronic migraine patients with symptoms of dry eye have shown a reduced fiber density on IVCM [27]. It has also been used to detect nerve damage and the reparative process in diabetics [28].

This in vivo imaging technique has recently been exploited to study the structural changes occurring in KC [29]. Morphological changes in the epithelium, the Bowman's layer, the subbasal, subepithelial, and stromal nerve plexus, keratocytes, collagen fibers, and the endothelial cells have been studied [29–31]. Fleischer ring and Vogt's striae are classically seen on the IVCM as hyper reflective structures [30]. Thus, it is also possible to study the specific morphological changes seen in the different layers of cornea in KC [31].

The subbasal nerve plexus has been mapped using the IVCM in KC with gross abnormal morphological changes even in patients with subclinical KC [9]. The plexus of nerves in KC shows a reduced nerve fiber density and increased tortuosity as compared to controls [32]. The functional effect of these changes has been established in a clinical study [33] where the authors have demonstrated reduced corneal sensitivity to different types of stimuli in patients with keratoconus. This suggests that though the impact of the disease process on the corneal nerves structurally and functionally has been described it has not been quantified as yet. Hence, we evaluated the subbasal plexus of nerves in a unique cohort of unilateral keratoconus where the differences between unaffected fellow eyes and affected keratoconic eyes were quantified and compared with controls. The utility of quantitatively detecting a subtle or early change to predict the disease onset or severity in the unaffected fellow eye is highlighted.

The quantification of the subbasal nerves was done by an automated software which extracts the nerve fiber data from a raw image thereby giving a “response” image which provides automated quantitative data regarding the CNFD, CNFL, CNBD, CTBD, CNFA, and CNFW. The analysis is objective, quick, and more reliable with negligible inter/intraobserver variability [34]. In our study, the quantitative analysis between the affected eye of unilateral KC (Group 2) and controls (Group 1) revealed a significant reduction in CNFD, CNBD, and CNFL, which is in accordance with previous reports [9]. In addition, there was a significant difference between the unaffected and affected eyes of the same individual. The values in the unaffected eyes were not similar to that in controls. This demonstrates the influence of the disease on topographically and clinically normal eyes. The subbasal nerve fiber quantitative changes might therefore help in establishing a diagnosis of KC in these eyes on follow-up even before it is manifested clinically. Besides serving as a disease marker it can also aid in monitoring disease progression.

There have been reports of subbasal nerve morphological changes at the base and apex of cones in KC. Subbasal nerves exhibit increased tortuosity with the branches running concentrically while following the contour of the topographic base of cone while the subbasal nerve fiber bundles at the apex have the most abnormal configurations which correlates well with ex vivo studies demonstrating that the greatest destruction of normal corneal architecture occurs at the apex of the cone [14]. We therefore also looked at the influence of the cone location in the KC group with 18 patients having a centered cone (Group 3a) and 15 patients with decentered cones (Group 3b). On analyzing the images taken at the apex of the cones in both subgroups, we found that all the parameters were increased in the decentered group with only the CNBD showing a statistically significant increase. Since we analyzed IVCM images taken only at the apex of the cornea, we hypothesize that the increased parameters in the decentered cone might be as a result of the base of cones being in the center of the cornea in contrast to central cones wherein the center of the cornea roughly corresponds to the apex of the cone. A quantification of the subbasal plexus of nerves and the correlation between parameters like CNFD, CNFL, and CNBD and grades of KC has also been reported [9].

At a cellular level, studies have implied that the abnormalities in keratoconus include the degeneration of epithelial basal cells and breaks in Bowman's layer, as well as the release of catabolic, proteolytic enzymes, and cytokines. This can potentially damage the corneal nerves and more particularly the Schwann cells passing between the acellular Bowman's layer and corneal epithelium [11, 12]. This is possibly the mechanism of morphological changes in the subbasal nerve plexus in established KC cases.

Currently there are no diagnostic modalities that can predict early changes prior to clinical manifestations or topographical changes. In our study we provide a quantitative analysis of the corneal subbasal nerve plexus in cases with unilateral KC with other risk factors for KC remaining constant for both eyes, allowing for a more objective comparison. In vivo confocal microscopy could be an extreme tool to provide insights into the pathogenesis of the disease and thereby influence the management strategy for each patient. Alterations in corneal nerve morphology can be used as an imaging marker for early diagnosis, for monitoring of progression, and for prognostication of keratoconus.

## Figures and Tables

**Table 1:** The mean of subbasal nerve plexus parameters with the standard deviation. Group 1 is control eye, Group 2 is unaffected eye of unilateral keratoconus, and Group 3 is keratoconic eye of unilateral keratoconus.

	Group 1 (*n* = 30)	Group 2 (*n* = 33)	Group 3 (*n* = 33)	Group 1 versus Group 2	Group 1 versus Group 3	Group 2 versus Group 3
CNFD	30.51 ± 5.8	28.48 ± 23.82	23.82 ± 8.02	0.26	<0.001	0.01
CNBD	43.45 ± 15.29	34.7 ± 16.1	37.61 ± 19.82	0.05	0.2	0.51
CNFL	17.59 ± 3.16	16.6 ± 2.42	14.82 ± 3.61	0.2	0.001	0.02
CTBD	59.73 ± 20.75	49.05 ± 25.3	54.66 ± 27.15	0.09	0.41	0.36
CNFA	0.01 ± 0.002	0.006 ± 0.001	0.006 ± 0.002	0.13	0.06	0.68
CNFW	0.02 ± 0.001	0.021 ± 0.002	0.02 ± 0.002	0.29	0.13	0.63

**Table 2:** The mean of subbasal nerve plexus parameters with the standard deviation. Group 3a is centered cones and group 3b is decentered cones.

	Centered (*n* = 18)	Decentered (*n* = 15)	*p* value
CNFD	21.6 ± 9.1	26.4 ± 5.6	0.09
CNBD	29.6 ± 18.9	47.1 ± 16.7	**<0.01**
CNFL	13.8 ± 4.3	15.9 ± 1.9	0.08
CTBD	46.9 ± 29.0	63.9 ± 22.1	0.07
CNFA	5 × 10^−3^ ± 2 × 10^−3^	6 × 10^−3^ ± 1 × 10^−3^	0.08
CNFW	2 × 10^−2^ ± 2 × 10^−3^	2 × 10^−2^ ± 1 × 10^−3^	1.0

## References

[1] Krachmer J. H., Feder R. S., Belin M. W. (1984). Keratoconus and related noninflammatory corneal thinning disorders. *Survey of Ophthalmology*.

[2] Bae G. H., Kim J. R., Kim C. H., Lim D. H., Chung E. S., Chung T.-Y. (2014). Corneal topographic and tomographic analysis of fellow eyes in unilateral keratoconus patients using pentacam. *American Journal of Ophthalmology*.

[3] Kennedy R. H., Bourne W. M., Dyer J. A. (1986). A 48-year clinical and epidemiologic study of keratoconus. *American Journal of Ophthalmology*.

[4] Holland D. R., Maeda N., Hannush S. B. (1997). Unilateral keratoconus. Incidence and quantitative topographic analysis. *Ophthalmology*.

[5] Li X., Rabinowitz Y. S., Rasheed K., Yang H. (2004). Longitudinal study of the normal eyes in unilateral keratoconus patients. *Ophthalmology*.

[6] Raiskup-Wolf F., Hoyer A., Spoerl E., Pillunat L. E. (2008). Collagen crosslinking with riboflavin and ultraviolet-A light in keratoconus: long-term results. *Journal of Cataract and Refractive Surgery*.

[7] Wittig-Silva C., Whiting M., Lamoureux E., Lindsay R. G., Sullivan L. J., Snibson G. R. (2008). A randomized controlled trial of corneal collagen cross-linking in progressive keratoconus: preliminary results. *Journal of Refractive Surgery*.

[8] Shetty R., Kaweri L., Pahuja N. (2015). Current review and a simplified ‘five-point management algorithm’ for keratoconus. *Indian Journal of Ophthalmology*.

[9] Bitirgen G., Ozkagnici A., Bozkurt B., Malik R. A. (2015). *In vivo* corneal confocal microscopic analysis in patients with keratoconus. *International Journal of Ophthalmology*.

[10] Niederer R. L., Perumal D., Sherwin T., McGhee C. N. J. (2008). Laser scanning in vivo confocal microscopy reveals reduced innervation and reduction in cell density in all layers of the keratoconic cornea. *Investigative Ophthalmology and Visual Science*.

[11] Shaheen B. S., Bakir M., Jain S. (2014). Corneal nerves in health and disease. *Survey of Ophthalmology*.

[12] Müller L. J., Vrensen G. F. J. M., Pels L., Cardozo B. N., Willekens B. (1997). Architecture of human corneal nerves. *Investigative Ophthalmology and Visual Science*.

[13] Morgan C. W., Nadelhaft I., de Groat W. C. (1978). Anatomical localization of corneal afferent cells in the trigeminal ganglion. *Neurosurgery*.

[14] Patel D. V., McGhee C. N. J. (2005). Mapping of the normal human corneal sub-basal nerve plexus by in vivo laser scanning confocal microscopy. *Investigative Ophthalmology & Visual Science*.

[15] Mathew J. H., Goosey J. D., Bergmanson J. P. G. (2011). Quantified histopathology of the keratoconic cornea. *Optometry and Vision Science*.

[16] Patel D. V., McGhee C. N. J. (2009). In vivo confocal microscopy of human corneal nerves in health, in ocular and systemic disease, and following corneal surgery: a review. *British Journal of Ophthalmology*.

[17] Shetty R., Nuijts R. M. M. A., Nicholson M. (2015). Cone location–dependent outcomes after combined topography-guided photorefractive keratectomy and collagen cross-linking. *American Journal of Ophthalmology*.

[18] Shetty R., D'Souza S., Srivastava S., Ashwini R. (2013). Topography-guided custom ablation treatment for treatment of keratoconus. *Indian Journal of Ophthalmology*.

[19] Abou Shousha M., Perez V. L., Fraga Santini Canto A. P. (2014). The use of Bowman's layer vertical topographic thickness map in the diagnosis of keratoconus. *Ophthalmology*.

[20] Sykakis E., Carley F., Irion L., Denton J., Hillarby M. C. (2012). An in depth analysis of histopathological characteristics found in keratoconus. *Pathology*.

[21] Sawaguchi S., Fukuchi T., Abe H., Kaiya T., Sugar J., Yue B. V. J. T. (1998). Three-dimensional scanning electron microscopic study of keratoconus corneas. *Archives of Ophthalmology*.

[22] Patel D. V., McGhee C. N. J. (2007). Contemporary in vivo confocal microscopy of the living human cornea using white light and laser scanning techniques: a major review. *Clinical and Experimental Ophthalmology*.

[23] Porzukowiak T. R., Ly K. (2015). In vivo confocal microscopy use in endotheliitis. *Optometry and Vision Science*.

[24] Parmar D. N., Awwad S. T., Petroll W. M., Bowman R. W., McCulley J. P., Cavanagh H. D. (2006). Tandem scanning confocal corneal microscopy in the diagnosis of suspected acanthamoeba keratitis. *Ophthalmology*.

[25] Brasnu E., Bourcier T., Dupas B. (2007). In vivo confocal microscopy in fungal keratitis. *British Journal of Ophthalmology*.

[26] Kheirkhah A., Darabad R. R., Cruzat A. (2015). Corneal epithelial immune dendritic cell alterations in subtypes of dry eye disease: a pilot in vivo confocal microscopic study. *Investigative Opthalmology & Visual Science*.

[27] Kinard K. I., Smith A. G., Singleton J. R. (2015). Chronic migraine is associated with reduced corneal nerve fiber density and symptoms of dry eye. *Headache*.

[28] Malik R. A., Kallinikos P., Abbott C. A. (2003). Corneal confocal microscopy: a non-invasive surrogate of nerve fibre damage and repair in diabetic patients. *Diabetologia*.

[29] Efron N., Hollingsworth J. G. (2008). New perspectives on keratoconus as revealed by corneal confocal microscopy. *Clinical and Experimental Optometry*.

[30] Uçakhan Ö. Ö., Kanpolat A., Ylmaz N., Özkan M. (2006). In vivo confocal microscopy findings in keratoconus. *Eye and Contact Lens*.

[31] Weed K. H., MacEwen C. J., Cox A., McGhee C. N. J. (2007). Quantitative analysis of corneal microstructure in keratoconus utilising in vivo confocal microscopy. *Eye*.

[32] Mannion L. S., Tromans C., O'Donnell C. (2005). An evaluation of corneal nerve morphology and function in moderate keratoconus. *Contact Lens and Anterior Eye*.

[33] Dienes L., Kiss H. J., Perényi K. (2015). Corneal sensitivity and dry eye symptoms in patients with keratoconus. *PLoS ONE*.

[34] Dabbah M. A., Graham J., Petropoulos I. N., Tavakoli M., Malik R. A. (2011). Automatic analysis of diabetic peripheral neuropathy using multi-scale quantitative morphology of nerve fibres in corneal confocal microscopy imaging. *Medical Image Analysis*.

